# Folding Free Energy Landscape of Ordered and Intrinsically Disordered Proteins

**DOI:** 10.1038/s41598-019-50825-6

**Published:** 2019-10-17

**Authors:** Song-Ho Chong, Sihyun Ham

**Affiliations:** Department of Chemistry, The Research Institute of Natural Sciences, Sookmyung Women’s University, Cheongpa-ro 47-gil 100, Yongsan-Ku, Seoul, 04310 Korea

**Keywords:** Computational chemistry, Biophysical chemistry, Computational biophysics

## Abstract

Folding funnel is the essential concept of the free energy landscape for ordered proteins. How does this concept apply to intrinsically disordered proteins (IDPs)? Here, we address this fundamental question through the explicit characterization of the free energy landscapes of the representative *α*-helical (HP-35) and *β*-sheet (WW domain) proteins and of an IDP (pKID) that folds upon binding to its partner (KIX). We demonstrate that HP-35 and WW domain indeed exhibit the steep folding funnel: the landscape slope for these proteins is ca. −50 kcal/mol, meaning that the free energy decreases by ~5 kcal/mol upon the formation of 10% native contacts. On the other hand, the landscape of pKID is funneled but considerably shallower (slope of −24 kcal/mol), which explains why pKID is disordered in free environments. Upon binding to KIX, the landscape of pKID now becomes significantly steep (slope of −54 kcal/mol), which enables otherwise disordered pKID to fold. We also show that it is the pKID–KIX intermolecular interactions originating from hydrophobic residues that mainly confer the steep folding funnel. The present work not only provides the quantitative characterization of the protein folding free energy landscape, but also establishes the usefulness of the folding funnel concept to IDPs.

## Introduction

Free energy landscape is the cornerstone in the study of protein folding. Its most fundamental aspect is that it is globally funneled such that the folding is energetically biased^1–3^. Indeed, this notion resolves the well-known paradox of Levinthal^4^, and accounts for why proteins fold in milliseconds to seconds instead of requiring astronomical timescales^5,6^. In recent years, the funneled landscape paradigm has been utilized also for understanding biomolecular binding as well as aggregation^7–9^. However, the usage of biomolecular free energy landscape has remained rather conceptual, which is in contrast to the quantitative role played by the potential energy surface in analyzing chemical reactions of small molecules. Herein, we develop a novel construction method for the protein free energy landscape to fill this gap. Pioneering works in this direction have been carried out through the density-of-state analysis of coarse-grained models^10^ and through the computation of enthalpy instead of free energy^11^. The method developed here can be distinguished from these previous works in that it is based on fully atomistic models for proteins and the direct evaluation of the free energy that defines the landscape^12,13^. We will apply this method to representative *α*-helical (HP-35^14^) and *β*-sheet (WW domain^15^) proteins to quantitatively argue the strength of the energetic bias toward the folded state.

Protein folding, on the other hand, does not always occur autonomously. In fact, the folding of numerous intrinsically disordered proteins, which is central to their functions, takes place only through the binding with their partners^16–18^. Can we understand the intrinsically disordered nature of a protein and rationalize its folding upon binding on the basis of the free energy landscape? This is the question we would like to address through the application of our construction method of the landscape. For this purpose, we investigate the pKID region of CREB protein, which is largely disordered when isolated, in the absence and presence of its binding partner, the KIX domain of CREB binding protein^19^. This is a well-studied paradigm that exhibits coupled folding and binding^20^. We aim to demonstrate that our explicit characterization of the landscape quantitatively captures common and distinctive features of ordered versus disordered proteins and that the folding funnel, which is steep enough for a disordered protein to fold, emerges as a result of the interaction with its binding partner.

Uncovering the molecular details of such an interaction underlying the folding upon binding of intrinsically disordered proteins is of fundamental importance in molecular biology and is of practical value in protein engineering. Site-directed mutagenesis is a powerful technique to probe effects on protein–protein interaction arising from specific amino acids in the sequence^21,22^. Related computational methods have also been developed such as computational alanine scanning of protein–protein interfaces^23^. These mutation-based approaches, however, necessarily invoke perturbations to the underlying protein structures, which sometimes exert disruptive effects in an unexpected and intricate manner^24^. Recently, we have developed a computational approach, termed the site-directed thermodynamic analysis method, that exactly decomposes protein thermodynamic functions into contributions from constituent amino acid residues^13,25–27^. Remarkably, this can be done without introducing any mutations, and our method is able to provide *in situ* characterization of protein–protein interaction at a detailed molecular level. By applying it to analyze the change in the pKID landscape induced by the binding with KIX, we will elucidate the detailed nature of the interaction relevant to the pKID–KIX coupled folding and binding.

## Results

### Constructing the folding free energy landscape

**Figure 1:**
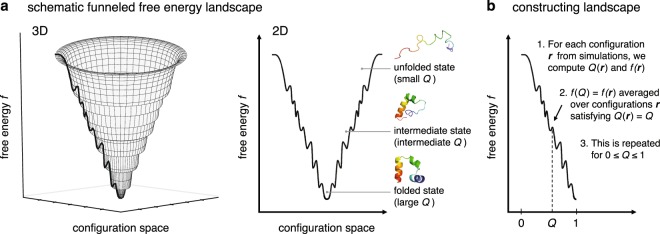
(**a**) Schematic representation in 3D (left panel) and 2D (right panel) of the funneled free energy landscape. (**b**) Steps to construct the landscape from all-atom simulations.

A typical diagram of the funneled free energy landscape is depicted in Fig. 1a, which schematically represents how the free energy decreases as the folding proceeds. To prepare for constructing such a diagram based on a fully microscopic approach, let us start from the precise definition of the landscape: it is the graph of the free energy $f({\bf{r}})$ expressed as a function of the positions (collectively abbreviated as **r**) of the atoms constituting a molecule of interest. Here, a molecule of interest is a protein, and all the rest of the system – surrounding water molecules and ions – is considered as solvent. The “free energy” *f* is then given by the gas-phase energy *E*_u_ and the solvation free energy ${G}_{{\rm{solv}}}$, $f({\bf{r}})={E}_{{\rm{u}}}({\bf{r}})+{G}_{{\rm{solv}}}({\bf{r}})$^12,13^. (The connection of *f* to the thermodynamic free energy will be presented below.) Since $f({\bf{r}})$ is defined over the high dimensional configuration space even for small proteins, one necessarily needs to resort to the dimensionality reduction to visualize and practically utilize the landscape. This can be done by introducing an order parameter (or reaction coordinate) *Q*, defined such that it takes small and large values respectively for the unfolded and folded states. The reduced landscape is then defined by $f(Q)$ which is the average of $f({\bf{r}})$ over a set of configurations $\{{\bf{r}}\}$ satisfying $Q=Q({\bf{r}})$.

Our method for the explicit construction of the landscape exactly follows what we just described (Fig. 1b). First, molecular dynamics simulations are performed that cover the protein’s unfolded and folded states. For each configuration **r** taken from the simulations, one computes $Q({\bf{r}})$ and $f({\bf{r}})$. The fraction of native amino acid contacts is chosen here as *Q*^28^. ${E}_{{\rm{u}}}({\bf{r}})$ in $f({\bf{r}})$ can easily be calculated from the force field parameters, and for ${G}_{{\rm{solv}}}({\bf{r}})$ we employ the molecular integral-equation theory (see Supplementary Methods). Based on $Q({\bf{r}})$ and $f({\bf{r}})$ for the simulated configurations, one can compute $f(Q)$ by averaging $f({\bf{r}})$ over those configurations having a specific $Q=Q({\bf{r}})$, and this is repeated for $0\le Q\le 1$. (This is illustrated in Supplementary Fig. S1 for HP-35.) The resulting $f(Q)$-versus-*Q* plot corresponds to the reduced free landscape with which we shall argue the landscape characteristics. It also provides the outline for constructing the 3D representation (see Fig. 1a,b), which will also be used in the following for the visualization purpose.

### Comments on the folding free energy landscape

Some comments might be in order here concerning the folding free energy landscape that we study in the present work. In the original work by Bryngelson *et al*.^1^, the concept of the folding funnel was introduced for the “energy landscape”. While an explicit expression was not given in that work, it was stated that the energy landscape is defined by “an effective free energy that is a function of the configuration of the protein to describe the protein–solvent system” and that “this description implicitly averages over the solvent coordinates”^1^. The explicit definition and derivation of the effective energy that defines the energy landscape can be found, e.g., in the article by Lazaridis and Karplus^12^: it is given by a sum of the gas-phase potential energy and the solvation free energy, that is, $f({\bf{r}})$ introduced above. We call $f({\bf{r}})$ the “free energy” since it includes the solvation free energy, and correspondingly, the energy landscape is referred to as the free energy landscape in the present work. The use of such a term for $f({\bf{r}})$ can be justified also by the fact that it is related to the probability distribution $P({\bf{r}})$ of observing a specific configuration **r** via $P({\bf{r}})\propto {e}^{-\beta f({\bf{r}})}$ with an inverse temperature $\beta =1/({k}_{{\rm{B}}}T)$. Finally, we notice that $f({\bf{r}})$ is defined for a single individual configuration **r**, and as such, it carries no configurational entropy.

It is important to recognize that the free energy landscape defined by $f({\bf{r}})$, as well as the reduced one introduced by $f(Q)$, are distinct from the free energy profile $F(Q)$ which is associated with the probability distribution $P(Q)$ of the order parameter *Q*, $F(Q)=-\,{k}_{B}T\,\log \,P(Q)$. In fact, these two free energies are related via $F(Q)=f(Q)-T{S}_{{\rm{config}}}(Q)$ in which ${S}_{{\rm{confg}}}(Q)$ is the configurational entropy^1,29^. $f(Q)$ and $F(Q)$ exhibit utterly different characteristics: While $f(Q)$ is globally funneled, i.e., there is an overall negative slope, toward the folded state, $F(Q)$ for a typical two-state folder shows the unfolded- and folded-state minima separated by a transition-state barrier. This is illustrated in Supplementary Fig. S2 displaying the $f(Q)$- and $F(Q)$-versus-*Q* curves for HP-35 and WW domain. Also, different computational approaches are necessary for $f(Q)$ and $F(Q)$. Indeed, whereas the sampling of equilibrium configurations is sufficient for constructing the free energy profile $F(Q)$, it is insufficient for obtaining the free energy landscape $f(Q)$: one also needs to quantify the solvation free energies of the individually sampled configurations^30^.

### Free energy landscapes for ordered versus disordered proteins

**Figure 2:**
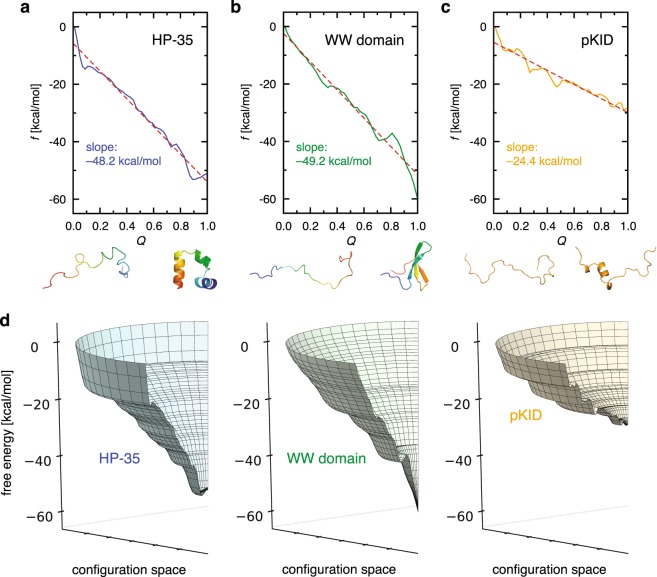
(**a**–**d**) Free energy landscapes for HP-35 (**a**), WW domain (**b**), pKID (**c**), and the comparison in 3-D representation (**d**). The free energy refers to the difference from the respective $f(Q=0)$, and the dashed line in (**a**–**c**) denotes a linear fit.

To extract common and distinctive characteristics of ordered and disordered proteins, we show and compare in Fig. 2 the free energy landscapes for HP-35, WW domain, and pKID. These landscapes were constructed based on their respective all-atom molecular dynamics simulations: for HP-35, we used the ~400 *μ*s folding-unfolding simulation trajectory^31^, and the *Q* and *f* values along the trajectory, necessary for constructing the landscape, are displayed in Supplementary Fig. S3; for WW domain, we used 6 independent simulation trajectories of 100 *μ*s^32,33^, and the *Q* and *f* values therefrom are shown in Supplementary Fig. S4; and for pKID, we conducted ~10 *μ*s molecular dynamics simulations, and the simulation results for the systems involving pKID are presented in Supplementary Figs S5 to S7. The simulations for HP-35 and WW domain were performed at close to their respective *in silico* melting temperatures, whereas those for pKID at 300 K. The force fields used were FF99SB*-ILDN^34–36^ for HP-35, FF99SB-ILDN^34,35^ for WW domain, and CHARMM22*^37–39^ for pKID; and the TIP3P water model^40^ was adopted for simulating all the systems. HP-35 and WW domain are respectively representative *α*-helical and *β*-sheet proteins, and pKID is a well-studied intrinsically disordered protein. We have chosen these particular systems also because their sequence lengths are comparable (HP-35 and WW domain, 35 residues; pKID, 34 residues): this suppresses sequence-length dependent effects that may obscure our analysis.

The overall slope of the landscape characterizes the global funneledness (the strength of the energetic bias) toward the folded state. The slope of the landscape for HP-35 estimated in Fig. 2a, −48.2 ± 1.7 kcal/mol, means that, e.g., 10% of the native contacts is formed with the free energy gain (decrease) by 4.8 kcal/mol. (The error estimation was done based on the block analysis as described in Supplementary Methods. We also computed the standard errors for the landscape curves, and the results are shown in Supplementary Fig. S8). Interestingly, the slope of the *β*-sheet WW domain (−49.2 ± 0.5 kcal/mol; see Fig. 2b) is found to be comparable to that of the *α*-helical HP-35. Such a degree of funneledness may be a typical one that is necessary to fold proteins of this sequence length (35 residues) against the unfavorable force arising from the configurational entropy. The landscape of disordered pKID, on the other hand, shows intriguing characters. Like HP-35 and WW domain, the overall landscape for pKID is somewhat funneled. However, the slope of the landscape for pKID is −24.4 ± 3.6 kcal/mol (see Fig. 2c) which is significantly smaller than that for HP-35 and WW domain. Such common and distinctive characteristics clearly show up in the 3D representation of the respective landscapes (Fig. 2d). Since the sequence lengths of HP-35, WW domain, and pKID are about the same, the magnitude of the unfavorable entropic force is expected to be comparable. The net driving force for folding is determined by a balance of the energetic bias, given by the slope of the landscape, and the opposing force arising from the configurational entropy, and the intrinsically disordered nature of pKID can be accounted for by the insufficient energetic bias to overcome the unfolding force for this sequence length. Thus, pKID is disordered not because the landscape is not funneled, but because the landscape is not steep enough to allow its folding.

### Binding-induced change in the landscape for pKID

**Figure 3:**
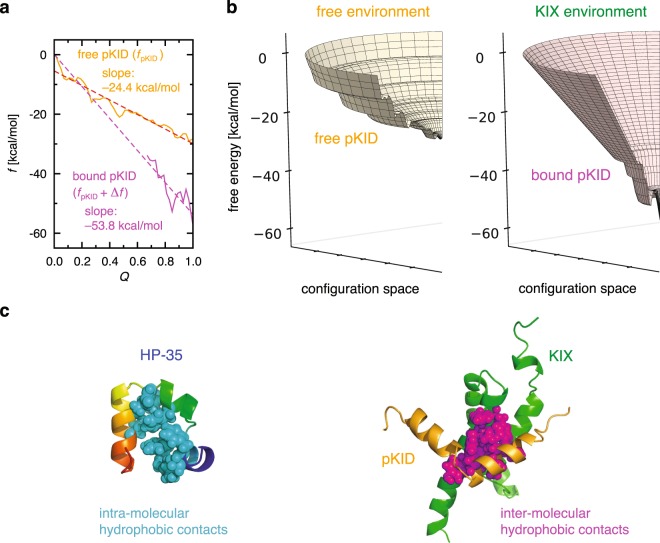
(**a**,**b**) Free energy landscape for the free pKID (colored orange) and the bound pKID (magenta) (**a**), and the comparison in 3-D representation (**b**). The free energy refers to the difference from the respective $f(Q=0)$, and the dashed line in (**a**) denotes a linear fit. (**c**) Amino acid residues forming intra-molecular hydrophobic contacts in HP-35 (PDB entry 1YRF) and inter-molecular hydrophobic contacts in pKID–KIX complex (PDB entry 2LXT) are represented by spheres.

pKID is also known as a paradigmatic disordered protein exhibiting the folding upon binding with its partner (KIX)^19,20^. To characterize this fascinating phenomenon in landscape terms, we investigate the change in the landscape of pKID induced by the binding. This can be done through a comparison of the landscape for the free pKID (free environment) and the one for the bound pKID in the pKID–KIX complex (KIX environment). The latter landscape can be constructed based on molecular dynamics simulations for the pKID–KIX complex. Here, the free energy needs to be extended to $f={f}_{{\rm{pKID}}}+\Delta {f}_{{\rm{int}}}$, which is a sum of the free energy for pKID, denoted as ${f}_{{\rm{pKID}}}$, and the solvent-averaged binding potential, $\Delta {f}_{{\rm{int}}}=\Delta {E}_{{\rm{int}}}+\Delta {G}_{{\rm{solv}}}$, incorporating the binding effect: $\Delta {E}_{{\rm{int}}}$ is the direct pKID–KIX interaction potential, and $\Delta {G}_{{\rm{solv}}}$ is the solvent-induced potential defined by ${G}_{{\rm{solv}}}({\rm{pKID}}:{\rm{KIX}})-[{G}_{{\rm{solv}}}({\rm{pKID}})+{G}_{{\rm{solv}}}({\rm{KIX}})]$^41,42^. The landscapes for the free and bound pKID are displayed in Fig. 3a,b. We find that the landscape for pKID gets significantly steeper upon binding, and its slope (−53.8 ± 12.9 kcal/mol) becomes comparable to that of HP-35 (−48.2 kcal/mol). Thus, the free energy landscape for pKID becomes steep in the KIX environment, and this provides the landscape explanation on why the binding with KIX is prerequisite for the folding of pKID.

### Site-directed analysis of the pKID–KIX interactions

It is thus the direct and solvent-mediated pKID–KIX interactions (both incorporated in $\Delta {f}_{{\rm{int}}}$) that confer the folding funnel on otherwise disordered pKID. Using the simulated pKID–KIX complex configurations, we computed the average $\Delta {f}_{{\rm{int}}}$ to be −25.4 kcal/mol. To further elucidate the molecular details of such interactions, we shall resort to the site-directed thermodynamic analysis method^13,25–27^. This method allows us to decompose $\Delta {f}_{{\rm{int}}}$ into contributions from individual constituent amino acid residues (see Supplementary Methods). To facilitate the understanding of our results, we will separately deal with neutral- and charged-residue contributions. In fact, we find that neutral residues provide more significant contributions ($\Delta {f}_{{\rm{int}}}^{{\rm{neutral}}}=-\,18.0\,{\rm{kcal}}/{\rm{mol}}$) than charged residues ($\Delta {f}_{{\rm{int}}}^{{\rm{charged}}}=-\,7.4\,{\rm{kcal}}/{\rm{mol}}$).

**Figure 4:**
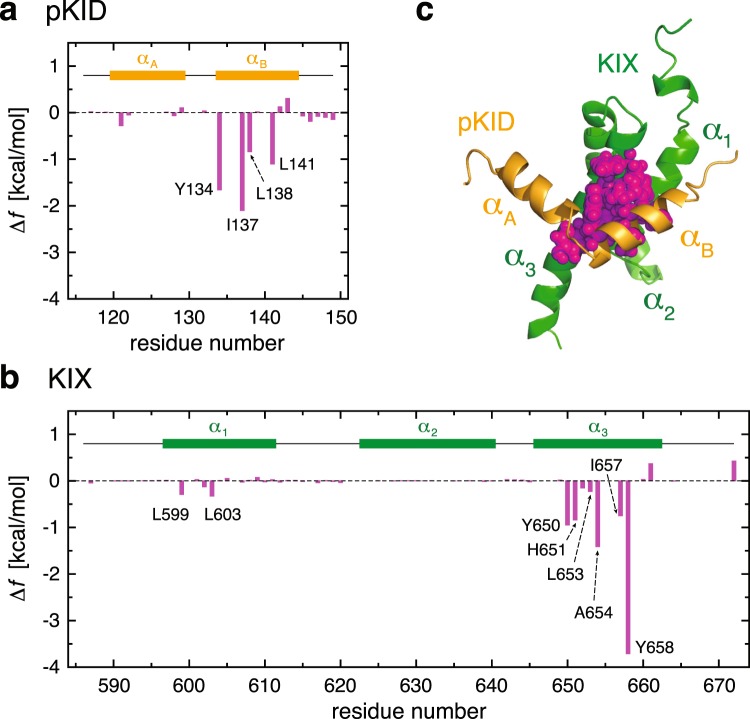
(**a**,**b**) Contributions to $\Delta {f}_{{\rm{int}}}$ from neutral residues of pKID (**a**) and KIX (**b**). (**c**) Amino acid residues forming inter-molecular hydrophobic contacts in pKID–KIX complex are represented by spheres.

Site-resolved contributions to $\Delta {f}_{{\rm{int}}}$ from neutral residues are shown in Fig. 4a,b, and the locations of the major contributing residues are displayed in Fig. 4c. We observe that major contributions arise from hydrophobic residues in the pKID *α*_B_ helix and those in the KIX *α*_3_ helix. In particular, Tyr-134 and Ile-137 provide the two largest contributions to $\Delta {f}_{{\rm{int}}}$ originating from pKID. This is in accord with the site-directed mutagenesis study, in which these two residues were found to be the most destabilizing residues in pKID when mutated to Ala^43^. Concerning the neutral residues in KIX, Tyr-658 and Ala-654 are the two most significant contributors to $\Delta {f}_{{\rm{int}}}$. The critical role of these residues in the pKID–KIX binding was discussed in the previous NMR study, and in particular, it was demonstrated that mutating Tyr-658 to Ala completely abolishes the complex formation^19^. Thus, our site-directed analysis method is able to identify those critical amino acid residues, and remarkably, this is achieved without introducing any mutations.

**Figure 5:**
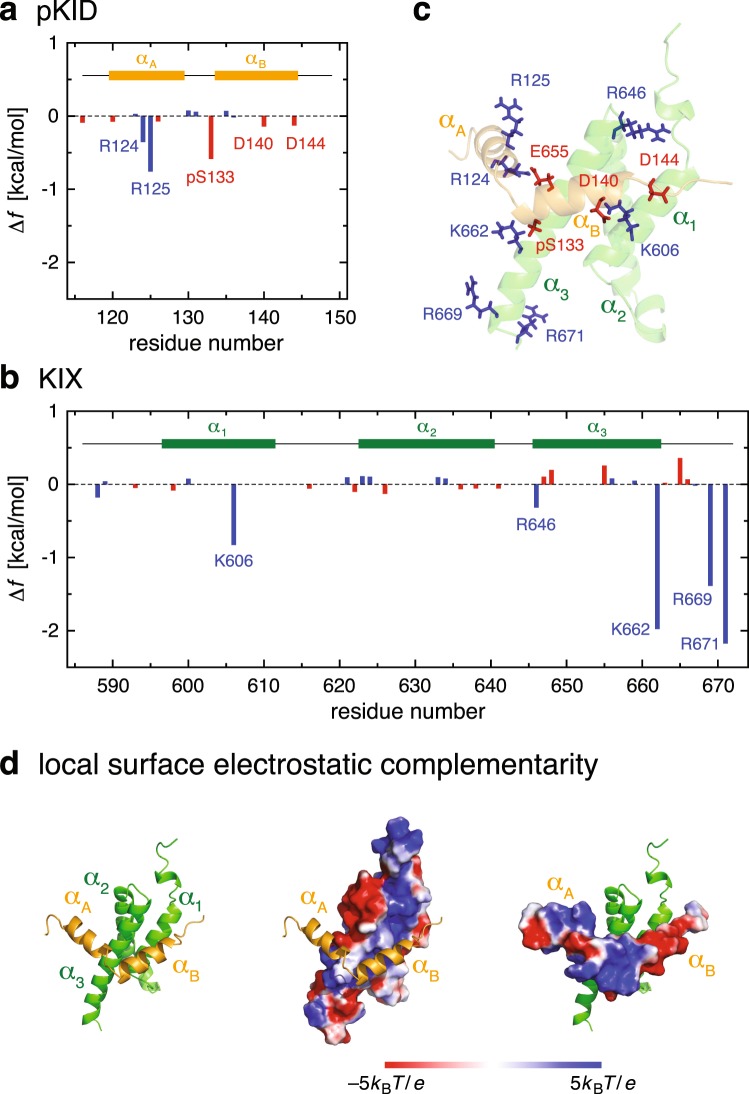
(**a**,**b**) Contributions to $\Delta {f}_{{\rm{int}}}$ from charged residues of pKID (**a**) and KIX (**b**). (**c**) Amino acid residues forming inter-molecular hydrogen bonds/salt bridges are indicate by stick representation. (**d**) Surfaces of KIX (middle panel) and pKID (right panel) are color coded by the electrostatic potential.

Site-resolved contributions to $\Delta {f}_{{\rm{int}}}$ arising from charged residues are displayed in Fig. 5a,b (see also Fig. 5c for their locations). One observes large negative contributions from Lys-662, Arg-669 and Arg-671 of KIX. To understand these results, we have analyzed representative inter-protein contacts involving charged residues, and the results are summarized in Supplementary Table S1. As listed there, the phosphoserine residue (pSer-133) of pKID forms a hydrogen bond to Lys-662 of KIX with a large population (~90%) and it is also hydrogen bonded to the C-terminal basic residues (Arg-669 and Arg-671) of KIX with substantial probabilities (~50% and ~70%, respectively). Thus, the favorable negative contributions to $\Delta {f}_{{\rm{int}}}$ from these residues reflect the presence of those stabilizing hydrogen-bond interactions between pKID and KIX.

We also find weak but non-negligible favorable contributions to $\Delta {f}_{{\rm{int}}}$ originating from Arg-124, Arg-125, Asp-140 and Asp-144 in pKID and from Lys-606 and Arg-646 in KIX (Fig. 5a,b). As can be inferred from Supplementary Table S1, these contributions are associated with the inter-protein contacts between oppositely charged residues. Motivated by this observation, we examined the surface electrostatic potential of pKID and KIX. Interestingly, we find alternating local electrostatic complementarity at the binding faces between the pKID *α*_A_ helix and the KIX *α*_3_ helix and between the pKID *α*_B_ helix and the other side of the KIX *α*_3_ helix (Fig. 5d): the binding side of *α*_A_ has positive electrostatic potential, which contacts with *α*_3_ having negative electrostatic potential; and the sign of electrostatic potential is reversed between *α*_B_ and the other side of *α*_3_. Since pKID must be docked with a proper position and orientation at the KIX surface in order to maximize such an interaction reflecting the local surface electrostatic complementarity, this weak interaction must be responsible for the binding specificity. Its relevance in the pKID–KIX binding is also corroborated by noticing that those amino acid residues listed above, as well as Glu-648 and Glu-655 in KIX generating negative surface potential for the binding with the pKID *α*_A_ helix, are well conserved in CREB and CBP family proteins^19^.

### Standard binding free energy

Finally, we argue the relation between the free energy *f* defining the landscape and the thermodynamic free energy. The free energy $f({\bf{r}})$ is defined for individual protein configurations **r**, and hence, it carries no configurational entropy. The thermodynamic free energy, on the other hand, is given by $F=-\,{k}_{B}T\,\log \,Z$ with $Z={\int }^{}\,d{\bf{r}}\,{e}^{-\beta f({\bf{r}})}$^12,13^. With the probability distribution, $P({\bf{r}})={e}^{-\beta f({\bf{r}})}/Z$, of observing a specific configuration **r**, and recalling the definition of the configurational entropy, ${S}_{{\rm{config}}}=-\,{k}_{B}\,{\int }^{}\,d{\bf{r}}\,P({\bf{r}})\,\log \,P({\bf{r}})$, one understands that *F* consists of an ensemble average of *f* and the configurational entropy, $F=\langle f\rangle -T{S}_{{\rm{config}}}$. For the binding thermodynamics, one additional term, called the external entropy (to be denoted as $\Delta {S}_{{\rm{ext}}}$), needs to be incorporated^44,45^. The standard binding free energy is then given by $\Delta {G}_{{\rm{bind}}}^{0}=\Delta \langle f\rangle -T(\Delta {S}_{{\rm{config}}}+\Delta {S}_{{\rm{ext}}})$^45^. Here, Δ*X* for $X=\langle f\rangle$ or ${S}_{{\rm{config}}}$ is given by ${X}_{{\rm{complex}}}-({X}_{{\rm{free}}{\rm{pKID}}}+{X}_{{\rm{free}}{\rm{KIX}}})$.

Using the simulated structures for the free pKID, free KIX, and pKID–KIX complex, we computed the terms that contribute to $\Delta {G}_{{\rm{bind}}}^{0}$ (see Supplementary Methods). The results of our computations, along with error estimations, are summarized in Supplementary Table S2. The resulting standard binding free energy, $\Delta {G}_{{\rm{bind}}}^{0}=-\,8.8\pm 11.8\,{\rm{kcal}}/{\rm{mol}}$, is in reasonable agreement with experiment (−8.1 kcal/mol)^46^. The large standard error of $\Delta {G}_{{\rm{bind}}}^{0}$ mainly comes from that of the configurational entropy term, $T\Delta {S}_{{\rm{config}}}$ (see Supplementary Table S2). In this regard, we notice that the magnitude of standard error is quite small (<1%) for $T{S}_{{\rm{config}}}$ of the three individual systems, but this is significantly enlarged when the difference ($T\Delta {S}_{{\rm{config}}}$) is taken because of the large cancellation of the individual contributions.

## Discussion

What could be the molecular origin of the different behavior between HP-35, an *α*-helical protein which autonomously folds, and pKID, which requires a partner for its folding into an *α*-helical structure? In this connection, we recall that a helical structure is in general not stable by itself, and additional stabilizing interactions must be present for its maintenance^47^. In fact, all the three *α* helices in HP-35 are tightly in contact with the hydrophobic core (left panel in Fig. 3c). On the other hand, intrinsically disordered proteins generally contain a low population of bulky hydrophobic residues^48,49^, and as such, pKID does not form intra-molecular hydrophobic contacts in the free environment. The presence/absence of the hydrophobic core in stabilizing the helical structure explains why the landscape for the free pKID is much shallower than that of HP-35. Upon the pKID–KIX binding, hydrophobic contacts can now be formed inter-molecularly (right panel in Fig. 3c), which contributes to stabilizing the helical structure of the pKID in KIX environment. The emergence of such additional intermolecular interactions upon binding renders the free energy landscape of the bound pKID to be steep enough to allow the folding of pKID.

Elucidating the molecular details of such interactions involving intrinsically disordered proteins is crucial to understand and eventually modify their function in gene regulation and signal transduction. While site-directed mutation is a common technique for identifying hot spots in protein–protein interactions, its application sometimes causes undesired significant alternations in protein structures. Here, we apply the site-directed thermodynamic analysis method – a computational approach that does not call for introducing any mutations – to provide *in situ* characterization of the pKID–KIX interactions. We find that interactions between hydrophobic residues that belong to the pKID *α*_B_ helix and the KIX *α*_3_ helix play a dominant role in the pKID–KIX complex formation. In particular, Tyr-134 and Ile-137 are found to be the most significant amino acid residues in pKID, and Ala-654 and Tyr-658 are the corresponding residues in KIX, which is in accord with the experimental observations^19,43^. We also show that positively charged residues in the pKID *α*_A_ helix and negatively charged residues in the KIX *α*_3_ helix provide weak but specific interactions between pKID and KIX.

Site-directed thermodynamic analysis thus reveals the presence of the strong interaction between the pKID *α*_B_ helix and the KIX *α*_3_ helix, which mainly arises from hydrophobic contacts, and of the weak but specific interaction between the pKID *α*_A_ helix and the other side of the KIX *α*_3_ helix, which is essentially of electrostatic origin. The presence of the two interactions that differ in strength will be responsible for the pKID–KIX binding process. In fact, it has been observed from the previous experimental studies that the binding of pKID to KIX involves an intermediate state where the transient complex is formed with the pKID *α*_B_ helix anchored to the KIX hydrophobic residues^20,43^. Computer simulation studies also observe the initial encounter complex formed by the docking of the pKID *α*_B_ helix to KIX, followed by the binding of the pKID *α*_A_ helix^50,51^. Our results on the pKID–KIX interactions explain such a sequence of events observed in the pKID–KIX binding process.

## Conclusions

Explicit characterization of the folding free energy landscape from fully microscopic approaches will significantly contribute to advancing our molecular-level understanding of protein folding phenomena. The present work develops a novel method for the explicit characterization based on atomistic simulations and the direct calculation of the free energy that defines the landscape. This method is applied to extract common and distinctive characteristics of the landscapes of ordered and intrinsically disordered proteins and to derive the landscape explanation on the folding upon binding. The method developed here is applicable to any atomistic simulations, and will be effective in expanding the scope of the funneled landscape perspective to a variety of processes that involve disordered proteins. We also apply the site-directed thermodynamic analysis method to provide detailed and *in situ* characterization of the interactions relevant to the coupled folding and binding. This analysis method identifies critical amino acid residues in protein–protein interactions without resorting to any mutations, and will also be valuable for identifying and characterizing hot spots in the protein–ligand interaction and the protein–DNA binding.

## Supplementary information

Supplementary Information
