# Supplementary material for: Folding Free Energy Landscape of Ordered and Intrinsically Disordered Proteins
Source: Sci Rep. 2019 Oct 17;9:14927. doi: 10.1038/s41598-019-50825-6 (PMC6797787; doi:10.1038/s41598-019-50825-6)
Supplement: Supplementary file 1 — Supplementary Information [file 41598_2019_50825_MOESM1_ESM.pdf]

Supplementary Information for

Folding Free Energy Landscape of Ordered and Intrinsically Disordered Proteins

Song-Ho Chong and Sihyun Ham*

Department of Chemistry, The Research Institute of Natural Sciences,
Sookmyung Women's University,
Cheongpa-ro 47-gil 100, Yongsan-Ku, Seoul 04310, Korea

Corresponding author:

*Sihyun Ham

Address Department of Chemistry
The Research Institute of Natural Sciences
Sookmyung Women's University
Cheongpa-ro 47-gil 100, Yongsan-Ku, Seoul 04310, Korea

Email sihyun@sookmyung.ac.kr

Phone +82-2-710-9410

Fax +82-2-2077-7321

Methods

Molecular dynamics simulations

For HP-35 and WW domain, we used all-atom simulation trajectories provided by the D. E. Shaw Research. These are folding-unfolding simulations carried out at close to their respective *in silico* melting temperatures, and the simulation of HP-35 consists of a single production run of ∼400 μs length[S1] and that of WW domain comprises 6 independent production runs of 100 μs length.[S2,S3]

For pKID and its complex with its binding partner KIX, we investigated the 34-residue form of the pKID region (residues 116–149) of CREB protein and the 87-residue construct of the KIX domain (residues 586–672) of the CREB binding protein. We carried out all-atom, explicit-water molecular dynamics simulations for free pKID, free KID, and pKID–KIX complex. (The difference between pKID and KID is in the phosphorylation at Ser-133, and the reason why we also conducted the free KID simulations becomes clear below.) The initial structures for these simulations were taken from the corresponding parts of the NMR complex structure (PDB entry 2LXT[S4]), which is actually a ternary complex consisting of pKID, KIX and another peptide referred to as MLL. All the simulations were done at T = 300 K and P = 1 bar using the pmemd.cuda module in AMBER16[S5] with the CHARMM22* force field[S6] for proteins and the TIP3P model[S7] for water. The CHAMBER utility[S8] was employed to use the CHARMM22* force field in AMBER16. While limitations have been reported of the CHARMM22* force field in describing intrinsically disordered proteins (see, e.g., ref. S9), its use for pKID can be justified according to our previous study.[S10] For example, the average α-helical contents of the α_A (residues 120 to 129) and α_B (residues 134 to 144) regions of free pKID from our simulations are $63.4 \pm 22.1\%$ and $9.0 \pm 14.3\%$, respectively, which are in fair agreement with the experimental observations ($\alpha_A \sim$ 50–60% and $\alpha_B \sim 15\%$). In addition, a successful folding of pKID was observed in our spontaneous, unguided pKID–KIX binding simulation that starts from an unfolded pKID.[S10]

The following common procedures were applied to conduct our simulations. The starting structure was solvated by water molecules and neutralizing counter ions in a cubic box with a buffer size of 15 Å. We first carried out the energy minimization of the system with harmonic restraints of a force constant of 500 kcal/(mol $Å^2$) applied to heavy atoms (1000 steps of steepest descent and 4000 steps of conjugate gradient minimizations) followed by the one without such restraints (5000 steps of steepest descent and 5000 steps of conjugate gradient minimizations). We then increased the system temperature from 0 K to 300 K with a 20 ps constant-volume simulation, followed by a 200 ps constant-pressure simulation at $P = 1$ bar using the Berendsen's method.[S11] These equilibration steps were repeated with different random initial velocities to perform independent production runs. The first 5 ns part of the production runs was done at constant pressure, and the rest at constant volume. Short-range nonbonded interactions were cut off at 12 Å, and electrostatic interactions were handled with the particle mesh Ewald method.[S12] We used the SHAKE algorithm[S13] to perform simulations with a 2 fs time step. Four independent 1 μs simulations were done for free pKID and free KID, and six independent 1 μs simulations for pKID–KIX complex.

Fraction of native contacts

We computed the fraction of native amino acid contacts (Q) following the procedure detailed in ref. S14 based on a list of native contact pairs. Native contact pairs refer to those heavy atom contact pairs – i atom in residue θ_i and j atom in residue θ_j separated by less than 4.5 Å and satisfying $|\,\theta_i - \theta_j\,| > 3$ – found in the native structure, which is usually taken from an X-ray or NMR study. We used the NMR structures for HP-35 (PDB entry 1YRF[S15]) and WW domain (2F21[S16]) to define native contact pairs in these systems, and the resulting Q versus simulation time is shown in Figs. S3 and S4.

On the other hand, we observe large deviations (>5 Å Cα root-mean-square fluctuations) between the simulated and NMR structures for pKID–KIX complex (Fig. S5). Such large conformational flexibility might be an intrinsic nature of the KIX domain that exhibits

binding promiscuity and cooperativity.[S17,S18] The large deviation might also be caused by the removal of the MLL peptide in our simulations, which is originally present in the NMR complex structure. Indeed, an inspection of Fig. S5 indicates that the removal of MLL significantly affects the KIX and pKID structures, which is in accord with the presence of allosteric communication between pKID and MLL through KIX.[S4] It is therefore inappropriate to adopt the NMR complex structure in defining the native contact pairs in pKID–KIX complex, and we instead used the simulated complex structures for this purpose. Rather than choosing a single representative structure, native contact pairs were defined based on those contact pairs whose population is >70 % during the equilibrium complex simulations. The resulting Q versus simulation time for free pKID and free KID is shown in Fig. S6 and that for pKID–KIX complex in Fig. S7.

Computation of the free energy

The free energy, $f = E_u + G_{\mathrm{solv}}$, that defines the free energy landscape is given by the gas-phase potential energy E_u and the solvation free energy G_{solv}. E_u can be calculated easily from the force field adopted in the simulations. For G_{solv}, rigorous computational methods are available based on the free energy perturbation or thermodynamic integration, but they are not suitable here since we need to calculate G_{solv} for quite a large number of simulated configurations. In the present work, we employed the the 3D-RISM (three-dimensional reference interaction site model) theory for its computation.[S19,S20] This is an integral-equation theory for obtaining the 3D distribution function $g_\gamma(\mathbf{r})$ of the water site γ at position $\mathbf{r}$ around the solute. In this theory, the distribution function is obtained by self-consistently solving the 3D-RISM equation

$$h_\gamma(\mathbf{r}) = \sum_{\gamma'} \int d\mathbf{r}' \, \chi_{\gamma\gamma'}(|\mathbf{r} - \mathbf{r}'|) \, c_{\gamma'}(\mathbf{r}') \tag{S1}$$

and the approximate closure relation

$$h_\gamma(\mathbf{r}) = \begin{cases} \exp[d_\gamma(\mathbf{r})] - 1 & \text{for} \quad d_\gamma(\mathbf{r}) \leq 0 \\ d_\gamma(\mathbf{r}) & \text{for} \quad d_\gamma(\mathbf{r}) > 0 \end{cases} \tag{S2}$$

in which $d_\gamma(\mathbf{r}) = -u_\gamma(\mathbf{r})/(k_\mathrm{B}T) + h_\gamma(\mathbf{r}) - c_\gamma(\mathbf{r})$. Here $h_\gamma(\mathbf{r}) = g_\gamma(\mathbf{r}) - 1$ and $c_\gamma(\mathbf{r})$ are the total and direct correlation functions, respectively; $\chi_{\gamma\gamma'}(r)$ denotes the site-site solvent susceptibility function which can be obtained either from simulations or integral-equation calculations; and $u_\gamma(\mathbf{r})$ is the solute-solvent interaction potential for a given solute configuration. We used the same numerical procedure as described in ref. S20 to solve the above equations. Solvation free energy can then be computed from the following analytical expression:

$$G_\mathrm{solv} = \rho k_\mathrm{B}T \sum_\gamma \int d\mathbf{r} \left[\frac{1}{2} h_\gamma(\mathbf{r})^2 \Theta(-h_\gamma(\mathbf{r})) - c_\gamma(\mathbf{r}) - \frac{1}{2} h_\gamma(\mathbf{r}) c_\gamma(\mathbf{r}) \right] \tag{S3}$$

Here, ρ is the average solvent number density, and Θ is the Heaviside step function.

The resulting free energy ($f = E_u + G_\mathrm{solv}$) versus simulation time for the systems we investigated is presented in Figs. S3, S4, S6 and S7.

Construction of the free energy landscape

We constructed the folding free energy landscapes ($f(Q)$-versus-Q plots) for HP-35, WW domain, and free pKID shown in Fig. 2 of the main text based on the Q- and f-versus-time data presented in Figs. S3, S4, S6 and S7, and this was done using the method illustrated in Fig. S1 (see the next subsection for more details on the construction of the landscape for pKID). The slope of the landscape was then estimated through a linear fit.

The error estimations for the landscape curves and the slopes therefrom were done based on the block analysis. For example, $\sim$400 μs simulation trajectory for HP-35 was first divided into 4 blocks of $\sim$100 μs length, the $f(Q)$-versus-Q plot was then constructed for each block and its slope was computed, and finally their standard errors were estimated: for WW

domain and pKID, the error estimations were done by regarding each of the independent simulation trajectories as a block. The standard errors for the free energy landscape curves are displayed in Fig. S8, and those for the slopes are reported in the main text.

Free energy landscape of disordered free pKID

The free energy landscape for free pKID is demonstrated as the cyan solid curve in the right panel of Fig. S6a. It is seen that only the $Q \gtrsim 0.4$ region is covered. This reflect the facts that (i) the free pKID simulations, initiated from the folded pKID structure taken from the NMR complex structure, are actually unfolding simulations since pKID when isolated is a disordered protein, and (ii) the pKID configurations sampled in the simulations are not completely unfolded and reach only up to $Q \sim 0.4$ as exemplified in the left panel of Fig. S6a. The presence of the residual structure in free pKID is in agreement with the experimental observation.[S21] To access the lower-Q region, we conducted the free KID simulations by "unphosphorylayting" the phosphorylated Ser-133 since experimentally it is known that free KID is more disordered than free pKID.[S21] Indeed, we find that configurations up to $Q \sim 0$ are sampled in the free KID simulations, and the resulting $f(Q)$-versus-Q plot covers the whole Q range (Fig. S6b). However, we cannot directly compare the free pKID and KID results because of the difference in free energy between the phosphorylated and unphosphorylated systems. To quantify the difference within the classical molecular mechanics, we proceeded as follows: (i) choose a free pKID configuration and compute its free energy $f_{\rm pKID}$, (ii) unphosphorylayte Ser-133 of that pKID configuration to obtain a KID configuration, and calculate its free energy $f_{\rm KID}$, (iii) compute the difference $f_{\rm pKID} - f_{\rm KID}$, and (iv) repeat this for 4000 free pKID configurations taken with a 1 ns interval from the four independent 1 μs simulation trajectories. As a result, we obtain the average difference $\pm$ standard deviation of -280.6 ± 6.1 kcal/mol. The $f(Q)$-versus-Q plots for free pKID and KID, in which the KID result is shifted to account for this difference, are compared in the right panel of Fig. S6a, and we find that their agreement is fairly well. The $f(Q)$-versus-Q plots for free pKID that

covers the whole Q range, shown in Fig. 2c of the main text, is obtained using both the free pKID and shifted KID results.

We remark that the use of the "shifted KID results" does not affect the main points mentioned in the main text concerning the free energy landscape of pKID. Indeed, just based the free pKID result shown as the cyan solid curve in the right panel of Fig. S6a, we obtain the slope of -17.6 kcal/mol characterizing the funneledness of the landscape, which remains close to the value (-24.4 kcal/mol) computed along with the shifted KID results. In particular, this does not alter the point that the landscape of free pKID is much less funneled than those of HP-35 and WW domain.

Site-directed thermodynamic analysis method

A computational method that provides an exact decomposition of the solvation free energy ($G_{\rm solv}$) into contributions from constituent amino acid residues ($G_{{\rm solv},i}$), $G_{\rm solv} = \sum_i G_{{\rm solv},i}$, has been developed in ref. S22. A corresponding partitioning can easily be derived for the gas-phase energy $E_{\rm u}$ for classical force fields, $E_{\rm u} = \sum_i E_{{\rm u},i}$. This is because E_u can in general be written as $E_{\rm u} = \sum_i \{E_i^{\rm intra} + (1/2)\sum_{j\neq i} E_{ij}^{\rm inter}\}$, where $E_i^{\rm intra}$ denotes the energy within the i-th residue and $E_{ij}^{\rm inter}$ the interaction energy between the i-th and j-th residues. (This separation into $E_i^{\rm intra}$ and $E_{ij}^{\rm inter}$ is obvious for the non-bonded terms such as the Lennard-Jones and Coulomb interactions, and can be done also for the bond terms. For example, the dihedral-angle potential involves four atoms, but these atoms belong to at most two residues. Therefore, the dihedral-angle potential can be classified into $E_i^{\rm intra}$ when all of those atoms belong to the same residue and into $E_{ij}^{\rm inter}$ otherwise.) By combining these results, one can decompose $f = E_{\rm u} + G_{\rm solv}$ into contributions from constituent amino acid residues (f_i), $f = \sum f_i$. The residue-resolved contributions to $\Delta f_{\rm int}$ can be obtained similarly.

Computation of the terms in the standard binding free energy

The statistical thermodynamic expression for the standard binding free energy is given by $\Delta G^0_{\mathrm{bind}} = \Delta\langle f\rangle - T(\Delta S_{\mathrm{config}} + \Delta S_{\mathrm{ext}})$.[S23] Here, ΔX for $X = \langle f\rangle$ or S_{config} represents the change in X upon complex formation, $X_{\mathrm{complex}} - (X_{\mathrm{free\,pKID}} + X_{\mathrm{free\,KIX}})$. We have already described our calculation method for f. The configurational entropy S_{confg} was computed using the energetic approach[S24,S25] according to which $TS_{\mathrm{config}} = \sigma_f^2/(2k_{\mathrm{B}}T)$ in terms of the variance σ_f^2 of f. For the external entropy ΔS_{ext}, we used the method developed in ref. S23 which requires six atoms for defining the external coordinates. Three from pSer-133 of pKID and three from Lys-662 of KIX, involved in the inter-protein hydrogen-bond of the largest population (see Table S1), were chosen for this purpose.

References

(S1) Piana, S.; Lindorff-Larsen, K.; Shaw, D. E. Protein Folding Kinetics and Thermodynamics from Atomistic Simulation. *Proc. Natl. Acad. Sci. U.S.A.* **2012**, *109*, 17845–17850.

(S2) Shaw, D. E.; Maragakis, P.; Lindorff-Larsen, K.; Piana, S.; Dror, R. O.; Eastwood, M. P.; Bank, J. A.; Jumper, J. M.; Salmon, J. K.; Shan, Y.; Wriggers, W. Atomic-Level Characterization of the Structural Dynamics of Proteins. *Science* **2010**, *330*, 341–346.

(S3) Piana, S.; Sarkar, K.; Lindorff-Larsen, K.; Guo, M.; Gruebele, M.; Shaw, D. E. Computational Design and Experimental Testing of the Fastest-Folding β-Sheet Protein. *J. Mol. Biol.* **2011**, *405*, 43–48.

(S4) Brüschweiler, S.; Konrat, R.; Tollinger, M. Allosteric Communication in the KIX Domain Proceeds through Dynamic Repacking of the Hydrophobic Core. *ACS Chem. Biol.* **2013**, *8*, 1600–1610.

(S5) Case, D. A. *et al. AMBER 16* (University of California, San Francisco, 2016).

(S6) Piana, S.; Lindorff-Larsen, K.; Shaw, D. E. How Robust Are Protein Folding Simulations with respect to Force Field Parameterization? *Biophys. J.* **2011**, *100*, L47–L49.

(S7) Jorgensen, W. L.; Chandrasekhar, J.; Madura, J. D.; Impey, R. W.; Klein, M. L. Comparison of Simple Potential Functions for Simulating Liquid Water. *J. Chem. Phys.* **1983**, *79*, 926–935.

(S8) Crowley, M. F.; Williamson, M. J.; Walker, R. C. CHAMBER: Comprehensive Support for CHARMM Force Fields within the AMBER Software. *Int. J. Quantum Chem.* **2009**, *109*, 3767–3772.

(S9) Robustelli, P.; Piana, S.; Shaw, D. E. Developing a Molecular Dynamics Force Field for Both Folded and Disordered Protein States. *Proc. Natl. Acad. Sci. U.S.A.* **2018**, *115*, E4758–E4766.

(S10) Chong, S.-H.; Im, H.; Ham, S. Explicit Characterization of the Free Energy Landscape of pKID–KIX Coupled Folding and Binding. *ACS Cent. Sci.* (2019). DOI: 10.1021/acscentsci.9b00200.

(S11) Berendsen, H. J. C.; Postma, J. P. M.; van Gunsteren, W. F.; DiNola, A.; Haak, J. R. Molecular Dynamics with Coupling to an External Bath. *J. Chem. Phys.* **1984**, *81*, 3684–3690.

(S12) Darden, T.; York, D.; Pedersen, L. Particle mesh Ewald: An N-log(N) Method for Ewald Sums in Large Systems. *J. Chem. Phys.* **1993**, *98*, 10089–10092.

(S13) Ryckaert, J.-P.; Ciccotti, G.; Berendsen, H. J. C. Numerical Integration of the Cartesian Equations of Motion of a System with Constraints: Molecular Dynamics of n-Alkanes. *J. Comput. Phys.* **1977**, *23*, 327–341.

(S14) Best, R. B.; Hummer, G.; Eaton, W. A. Native Contacts Determine Protein Folding Mechanisms in Atomistic Simulations. *Proc. Natl. Acad. Sci. U.S.A.* **2013**, *110*, 17874–17879.

(S15) McKnight, C. J.; Matsudaira, P. T.; Kim, P. S. NMR Structure of the 35-Residue Villin Headpiece Subdomain. *Nat. Struct. Biol.* **1997**, *4*, 180–184.

(S16) Jäger, M.; Zhang, Y.; Bieschke, J.; Nguyen, H.; Dendle, M.; Bowman, M. E.; Noel, J. P.; Gruebele, M.; Kelly, J. W. Structure–Function–Folding Relationship in a WW Domain. *Proc. Natl. Acad. Sci. U.S.A.* **2006**, *103*, 10648–10653.

(S17) Goto, N. K.; T. Zo and, M. M.-Y.; Dyson, H. J.; Wright, P. E. Cooperativity in

Transcription Factor Binding to the Coactivator CREB-Binding Protein (CBP). *J. Biol. Chem.* **2002**, *277*, 43168–43174.

(S18) Thakur, J. K.; Yadav, A.; Yadav, G. Molecular Recognition by the KIX Domain and its Role in Gene Regulation. *Nucleic Acids Res.* **2014**, *42*, 2112–2125.

(S19) Kovalenko, A. In *Molecular Theory of Solvation*; Hirata, F., Ed.; Kluwer Academic: Dordrecht, 2003; pp 169–275.

(S20) Imai, T.; Harano, Y.; Kinoshita, M.; Kovalenko, A.; Hirata, F. A Theoretical Analysis on Hydration Thermodynamics of Proteins. *J. Chem. Phys.* **2006**, *125*, 024911.

(S21) Radhakrishnan, I.; Pérez-Alvarado, G. C.; Dyson, H. J.; Montminy, M. R.; Wright, P. E. Conformational Preferences in the Ser^{133}-Phosphorylated and Non-Phosphorylated Forms of the Kinase Inducible Transactivation Domain of CREB. *FEBS Lett.* **1998**, *430*, 317–322.

(S22) Chong, S.-H.; Ham, S. Atomic Decomposition of the Protein Solvation Free Energy and Its Application to Amyloid-Beta Protein in Water. *J. Chem. Phys.* **2011**, *135*, 034506.

(S23) Chong, S.-H.; Ham, S. New Computational Approach for External Entropy in Protein–Protein Binding. *J. Chem. Theory Comput.* **2016**, *12*, 2509–2516.

(S24) Chong, S.-H.; Ham, S. Configurational Entropy of Protein: A Combined Approach Based on Molecular Simulation and Integral-Equation Theory of Liquids. *Chem. Phys. Lett.* **2011**, *504*, 225–229.

(S25) Chong, S.-H.; Ham, S. Dissecting Protein Configurational Entropy into Conformational and Vibrational Contributions. *J. Phys. Chem. B* **2015**, *119*, 12623–12631.

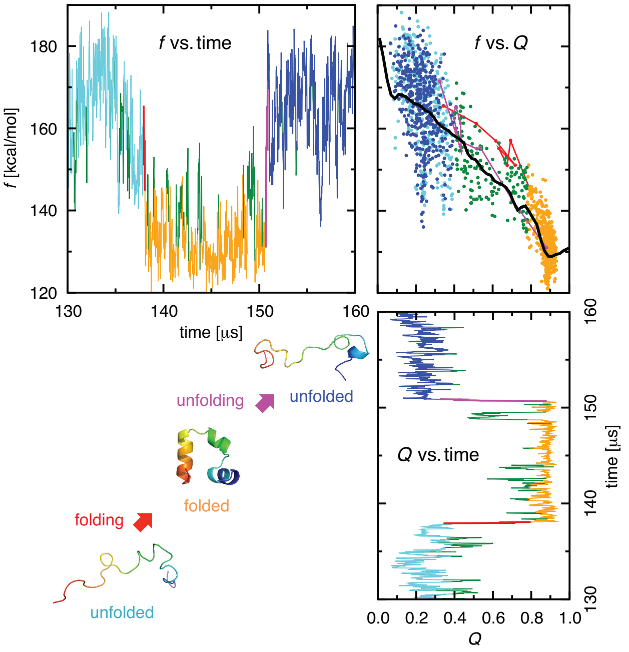

Figure S1: Illustration of the construction of the free energy landscape using the ∼400 μs folding-unfolding simulation trajectory of an α-helical protein (HP-35) provided by the D. E. Shaw Research.[S1] The f and Q values for the configurations along a selected part of the trajectory are respectively shown in the left-upper and right-bottom panels: those values along the whole ∼400 μs trajectory are presented in Fig. S3. One observes from the Q-versus-time plot that a folding (change in Q from small to large values; the time region colored red) and an unfolding (opposite change in Q; colored magenta) occur respectively once in this part of the trajectory. Corresponding variations are also discernible in the f-versus-time plot: the folding/unfolding is associated with the decrease/increase in f, respectively. The average $f(Q)$-versus-Q plot can then be constructed from a scatter plot displayed in the right-upper panel, and the solid line added in this panel refers to the average based on the whole ∼400 μs trajectory.

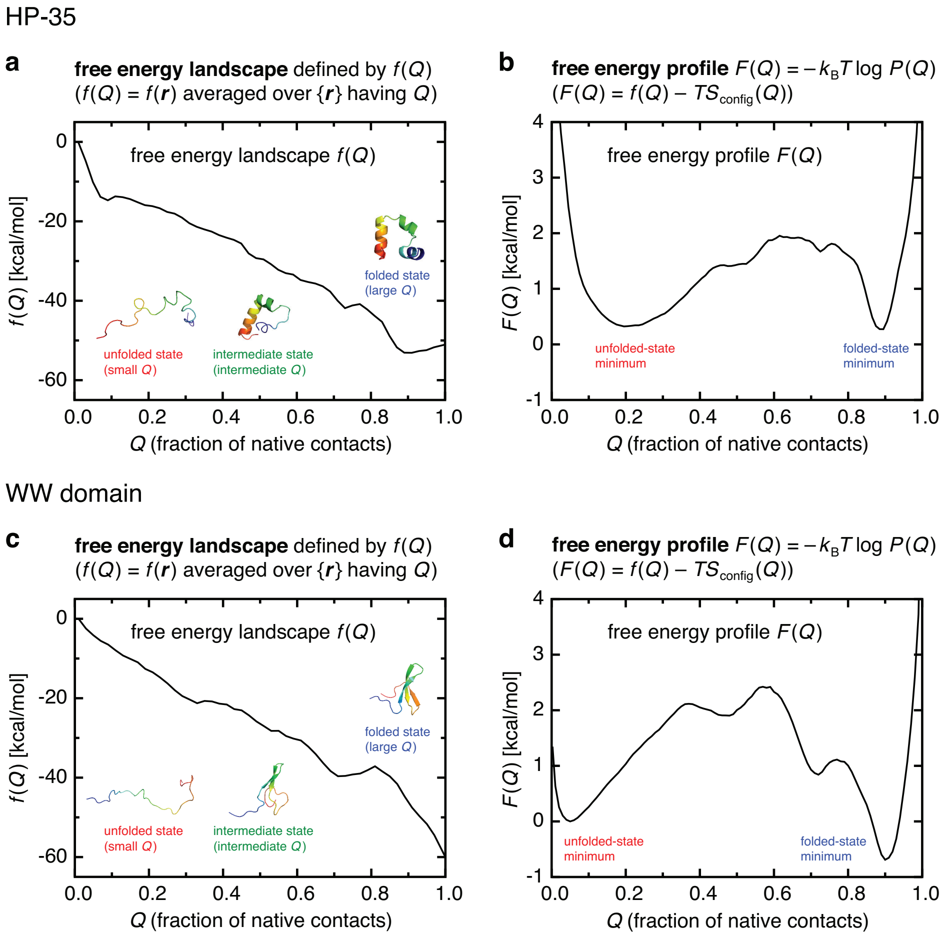

Figure S2: (a,b) Fee energy landscape defined by $f(Q)$ (a) and the free energy profile $F(Q)$ based on the $\sim$ 400 μs folding–unfolding simulation trajectory of HP-35. (c,d) Corresponding results based on the six independent 100 μs simulation trajectories of WW domain.

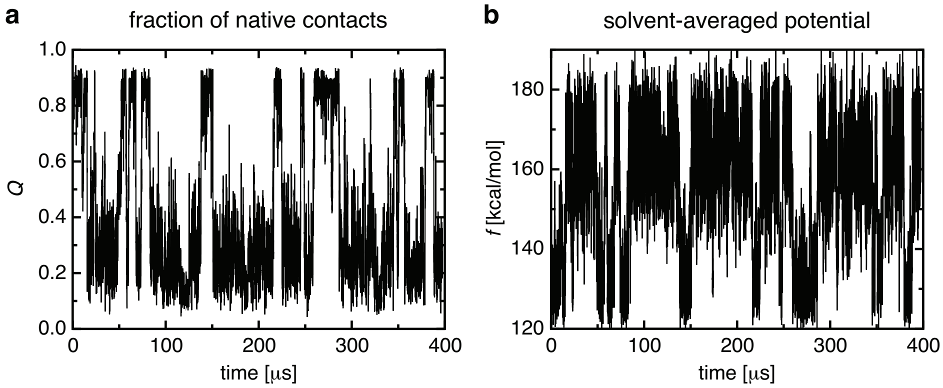

Figure S3: (a,b) Fraction of native contacts Q (a) and free energy f (b) versus simulation time for the whole ∼400 μs simulation trajectory of HP-35.

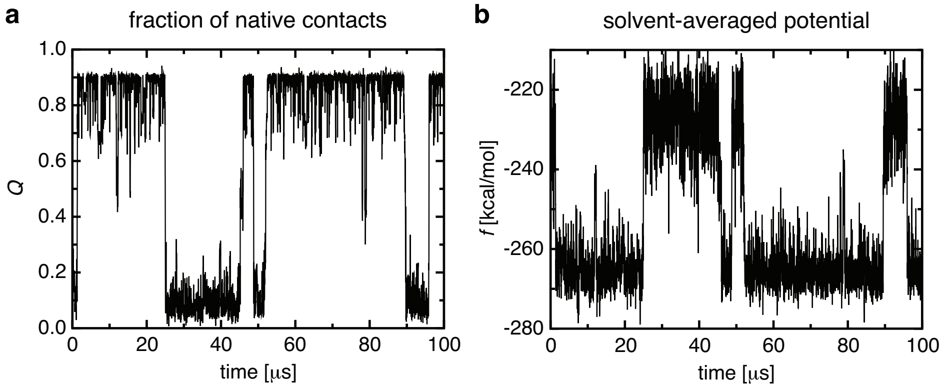

Figure S4: (a,b) Fraction of native contacts Q (a) and free energy f (b) versus simulation time for one of the six independent 100 μs simulation trajectories of WW domain.

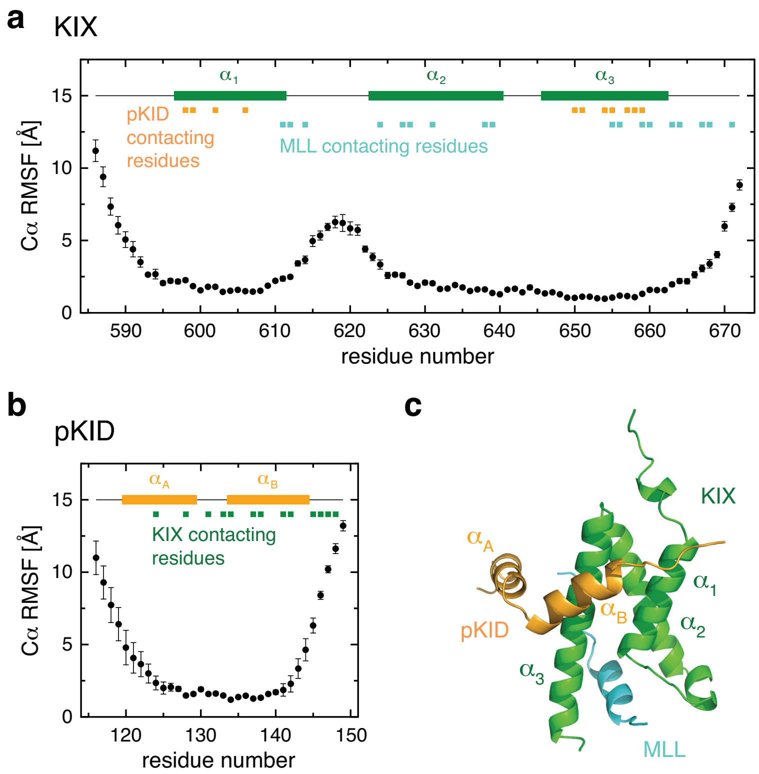

Figure S5: (a,b) Cα root-mean-square fluctuations (RMSF) relative to the NMR complex structure for KIX (a) and pKID (b). Averages (filled circles) and standard errors (bars) were computed from the six independent simulation trajectories of the pKID–KIX complex. Locations of the alpha helices (horizontal bars) and contacting residues (squares) are indicated in each panel. (c) NMR complex structure of PDB entry 2LXT[S4] consisting of pKID, KIX and MLL. The removal of MLL in our complex simulations affects those residues in KIX which are making contacts with MLL in the NMR complex structure (marked with cyan squares). This will in turn affect RMSF values of the pKID residues.

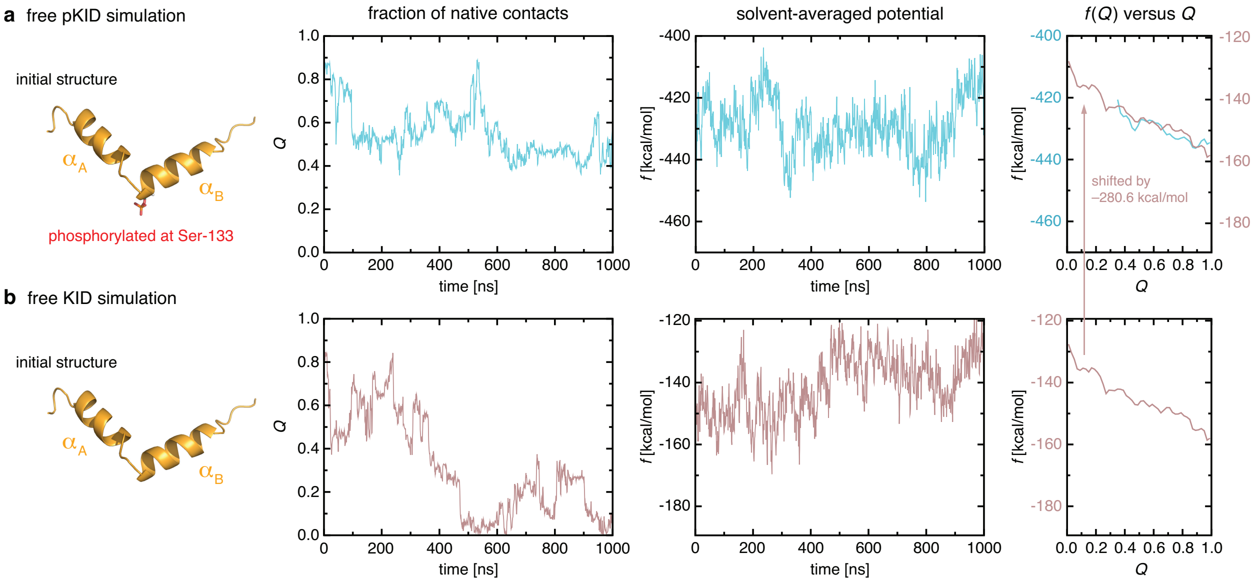

Figure S6: (a,b) Fraction of native contacts Q (left panel) and free energy f (middle panel) versus simulation time and the average $f(Q)$ versus Q (right panel) for free pKID (a) and free KID (b) based on one of the respective four independent 1 μs simulation trajectories.

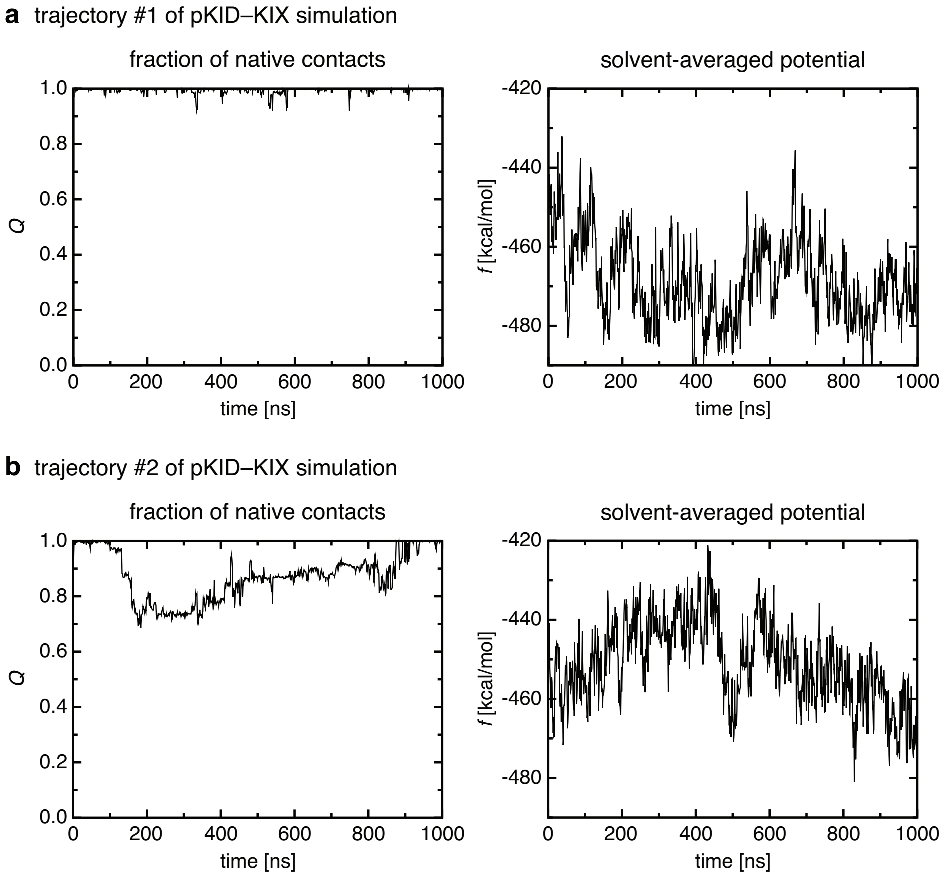

Figure S7: (a,b) Fraction of native contacts Q and free energy f versus simulation time for trajectory #1 (a) and trajectory #2 (b) of the six independent 1 μs simulation trajectories of the pKID–KIX complex.

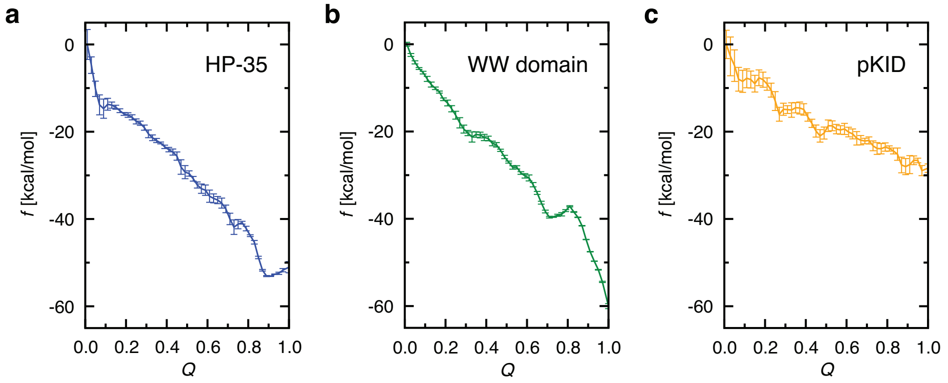

Figure S8: (a–c) Free energy landscape curves (solid lines) along with the standard errors (vertical bars) for HP-35 (a), WW domain (b), and pKID (c).

Table S1: Population (%) of charged-residue contacts at the pKID–KIX binding interface[a]

pKID (helix ID)	KIX (helix ID)	heavy-atom contact[b]	hydrogen-bond/salt-bridge[c]
R124 (α_A)	E655 (α_3)	61.2 ± 10.4	57.7 ± 10.7
R125 (α_A)	E648 (α_3)	81.3 ± 5.6	63.2 ± 9.2
R125 (α_A)	H651 (α_3)	94.2 ± 4.3	33.9 ± 12.1
pS133	Y658 (α_3)	96.8 ± 1.1	49.4 ± 16.2
pS133	K662 (α_3)	90.4 ± 3.9	89.3 ± 4.1
pS133	R669	46.5 ± 14.2	45.9 ± 14.2
pS133	R671	71.5 ± 6.0	71.5 ± 6.0
Y134 (α_B)	H651 (α_3)	98.9 ± 0.7	60.0 ± 12.4
D140 (α_B)	K606 (α_1)	87.8 ± 4.1	67.5 ± 6.9
D144 (α_B)	R646 (α_3)	35.7 ± 7.4	27.4 ± 6.0

[a] Average ± standard error. [b] A heavy-atom contact is considered formed between two residues if the minimum heavy-atom distance is lower than 4.5 Å. [c] A hydrogen-bond is considered formed if the minimum O-O, O-N or N-N distance is lower than 3.5 Å. A salt-bridge refers to a hydrogen-bond formed with side chains of charged residues.

Table S2: Binding thermodynamic quantities (kcal/mol)[a]

	$\langle f \rangle$	TS_{config}	$G = \langle f \rangle - TS_{\text{config}}$	
free pKID	-432.0 ± 1.0	336.2 ± 2.2	-768.2 ± 1.4	
free KIX	87.1 ± 2.3	881.0 ± 4.6	-793.9 ± 5.0	
pKID–KIX complex	-370.2 ± 1.9	1206.9 ± 11.2	-1577.1 ± 10.6	
difference	$\Delta\langle f \rangle$	$T\Delta S_{\text{config}}$	$\Delta\langle f \rangle - T\Delta S_{\text{config}}$	$T\Delta S_{\text{ext}}$
	-25.4 ± 3.1	-10.4 ± 12.3	-15.0 ± 11.8	-6.2 ± 0.2
ΔG^0_{bind}				$\Delta G - T\Delta S_{\text{ext}}$
				-8.8 ± 11.8

[a] Average ± standard error.
